# Development and Validation of Sex-Specific Markers in *Pelodiscus Sinensis* Using Restriction Site-Associated DNA Sequencing

**DOI:** 10.3390/genes10040302

**Published:** 2019-04-15

**Authors:** Hongwei Liang, Lihua Wang, Hang Sha, Guiwei Zou

**Affiliations:** 1 Yangtze River Fisheries Research Institute, Chinese Academy of Fishery Sciences, Wuhan 430223, China; lihuawang@yfi.ac.cn (L.W.); ikriss@163.com (H.S.); 2 Key Laboratory of Aquatic Animal Genomics, Ministry of Agriculture, Chinese Academy of Fishery Sciences, Wuhan 430223, China

**Keywords:** sex-specific marker, RAD-seq, genome walking, sex identification, *Pelodiscus sinensis*

## Abstract

The sex of an animal influences its economic traits, especially in species displaying sexual dimorphism. The Chinese soft-shelled turtle, *Pelodiscus sinensis*, is an economically important aquatic species that shows significant male sexual dimorphism, with a large body size, faster growth, a thick and wide calipash, and lower body fat. In this study, ten male and ten female turtles were subjected to restriction site-associated DNA sequencing (RAD-seq) using the Hi-Seq 4000 sequencing platform to isolate female-specific DNA fragments. We identified 5967 bp and 6532 bp fragments using genome walking. Three female-specific markers designed from these two fragments were confirmed to separate the sexes of *Pelodiscus sinensis* perfectly. One of the female-specific markers showed dosage association in female and male individuals. Individuals from different populations (n = 296) were used to validate that the female-specific markers could identify the genetic sex of *Pelodiscus sinensis* with 100% accuracy. The results of the present study demonstrated that RAD-seq was useful to develop sex-related markers in animals, and verified that the sex determination system of *Pelodiscus sinensis* belonged to the ZZ/ZW heterogametic system. Importantly, the developed markers could lead to a method for sex-controlled breeding in the Chinese soft-shelled turtle.

## 1. Introduction

An animal’s genetic sex influences its economic traits, especially in animals displaying significant sexual dimorphism. The Chinese soft-shelled turtle, *Pelodiscus sinensis*, is widely distributed in China, Russia, Japan, Thailand, Vietnam, and Korea [1,2]. It is an economically important aquatic species, and in China, it is the most common turtle species used for food and medicine or sold as a pet [3]. Aquaculture practices for the Chinese soft-shelled turtle have revealed significant sexual dimorphism, in which the male individuals are characterized by a larger body size, faster growth, a thicker and wider calipash, and less body fat compared with female turtles. Therefore, juvenile male turtles are more popular in the market. This sexual dimorphism has encouraged research to produce a high-male rate or all-male juveniles using sex control approaches. 

Sex-specific or sex chromosome-linked markers have significant applications for the genetic improvement of economically important traits in aquaculture species [4,5], especially for the uncommon growth differences between females and males [6]. Sex-specific or sex chromosome-linked markers make it possible to select superior individuals or exclude unwanted individuals at the DNA level, and can be used to increase the efficiency and precision of selecting individuals that have desired genetic traits at an early stage [7]. In addition, sex-specific markers can reveal whether a species has genetic sex determination (GSD) with either male or female heterogamety [8]. Random amplified polymorphic DNA fingerprinting (RAPD), microsatellite markers (SSR), amplified fragment length polymorphism (AFLP), or restriction site-associated DNA sequencing (RAD-seq) have been successfully used to obtain sex-specific markers of more than fifteen economically important aquatic species [9,10,11,12,13,14]. 

Sex determination in the Chinese soft-shelled turtle has been described using the ZZ/ZW heterogametic system [15,16]. However, to date, no sex-specific or sex chromosome-linked markers have been developed, which has seriously limited the development of gender control technology in this species. Therefore, the aim of the present study was to develop sex-specific genetic markers using RAD-seq, and to use the developed markers for genetic sex identification in Chinese soft-shelled turtles from different locations. 

## 2. Materials and Methods

### 2.1. Animals and DNA Extraction 

Ten male and ten female farmed Chinese soft-shelled turtles (named PSM1 to PSM10, and PSF1 to PSF10) were collected from Anhui Xijia Agricultural Development Co. Ltd (Bengbu, China). The calipash tissues were sampled, immersed in absolute ethanol, and stored at −20 °C. Genomic DNA was isolated from the alcohol-preserved calipash tissues using a DNA extraction kit (Takara, Shiga, Japan), according to the manufacturer’s protocol. The DNA quality and concentration were evaluated using 1.0% agarose electrophoresis, measured using a NanoDrop 2000 spectrophotometer (Thermo scientific, Waltham, MA, USA), and then stored at −20 °C. All procedures using the experimental turtles conformed to the standards for Animal Care of the Yangtze River Fisheries Research Institute, Chinese Academy of Fishery Sciences (Wuhan, China).

### 2.2. Restriction Site-Associated DNA Sequencing 

RAD-seq-reduced representation libraries for each sample were constructed following a previously described protocol, with some modifications [17]. Briefly, the high-fidelity restriction enzyme *EcoRI* (specific recognition sequence: G|AATTC, New England Biolabs, Ipswich, MA, USA) was used to digest the genomic DNA (about 1 µg) from each sample at 65 °C for 20 min. Individually barcoded P1 adapters were ligated onto the *EcoRI* cut site in each sample. A total of 20 samples were constructed (PSF1 to PSF10, and PSM1 to PSM10). Then, 150 bp paired-end (PE) sequencing libraries were constructed for the 20 samples. Each individual sample was sequenced in different lanes using the IIIumina Hiseq 4000 platform (IIIumina, San Diego, CA, USA). The RAD-seq library construction and sequencing were performed by the Gene Denovo Biotechnology Co. (Guangzhou, China).

### 2.3. RAD-seq Data Analysis 

To improve the quality of the raw sequence reads, the Illumina sequencing reads were trimmed, and low-quality bases were removed before the analysis. High-quality clean reads were obtained using the following three steps: First, reads with an unidentified nucleotides (N) percentage >10% were removed; second, low-quality reads with >50% of bases having a phred quality score of ≤20 were discarded; and third, reads with only barcode adapters were removed. The high-quality reads were then used for further analysis. The filtered reads of each female and male individual were mapped to the Chinese soft-shelled turtle reference genome sequence (PelSin_1.0, https://www.ncbi.nlm.nih.gov/assembly/GCF_000230535.1) using the Burrows–Wheeler Aligner (BWA) software with the parameter “mem 4-k32-M”, where -k is the minimum seed length, and –M is an option used to mark shorter split alignment hits as secondary alignments [18]. Based on the alignment result, the common reads between the PSF and PSM samples were removed. The sex determination of the Chinese soft-shelled turtle has been described as a ZZ/ZW heterogametic system; therefore, the collected scaffolds were filtered to identify those with >100 total reads in all ten female samples, but <10 in total reads in all ten male samples. Finally, the scaffold sequences satisfying the above criteria were selected as candidate female-specific regions.

### 2.4. Sex-Specific Markers Identification

Based on the different candidate regions, the top 10 different sequences were used to design PCR primers for putative sex-specific marker region identification, using Primer Premier 5.0 software (http://www.premierbiosoft.com/primerdesign/) (Table 1). The PCR reaction volume of 25 µL contained 2.5 μL of 10× PCR buffer mix (TsingKe, Beijing, China), 0.5 μL of 10 μM of each primer, 0.5 μL of 5 U/μL *Taq* DNA polymerase (Takara, Japan), 1 μL of 50 ng/μL DNA, and 20 μL of ultrapure water. The PCR amplification conditions were as follows: Pre-denaturation at 94 °C for 5 min; denaturation at 94 °C for 30 s, 30 s at the denaturation temperature of the different primers, and 45 s at 72 °C, for 32 cycles; and a final extension at 72 °C for 4 min. The amplification products were then detected using a 1.5% agarose gel. 

In addition, for the specific sequences that displayed different results in male and female populations, but without remarkably different agarose gel bands, the genome walking method was performed using a Universal GenomeWalker 2.0 kit (Clontech, Mountain View, CA, USA) to obtain their longer sequences to design primers. Then, the GenomeWalker PCR products were sequenced, aligned, and assembled using the software DNAMAN (Lynnon Biosoft, San Ramon, CA, USA). Thereafter, the sex-specific regions were used to design sex-specific markers.

### 2.5. Validation of Sex-Specific Markers 

To validate the putative sex-specific primers, two farmed populations of the Chinese soft-shelled turtle were collected. One population, comprising 48 female and 48 male individuals, was sampled from Xiantao, Hubei province, and the other, comprising 100 female and 100 male individuals, came from Hanshou, Hunan province. All 296 turtles were adults; therefore, their morphology was macroscopically observed or they were dissected to detect the gonad tissue to determine their sex. Genomic DNA was extracted from the female and male calipash tissues, and PCR was used to amplify the sex difference region to detect their genetic sex. Finally, their sex was cross checked with that detected using the gonad tissue.

## 3. Results 

### 3.1. Restriction Site-Associated DNA Sequencing 

**Figure 1:**
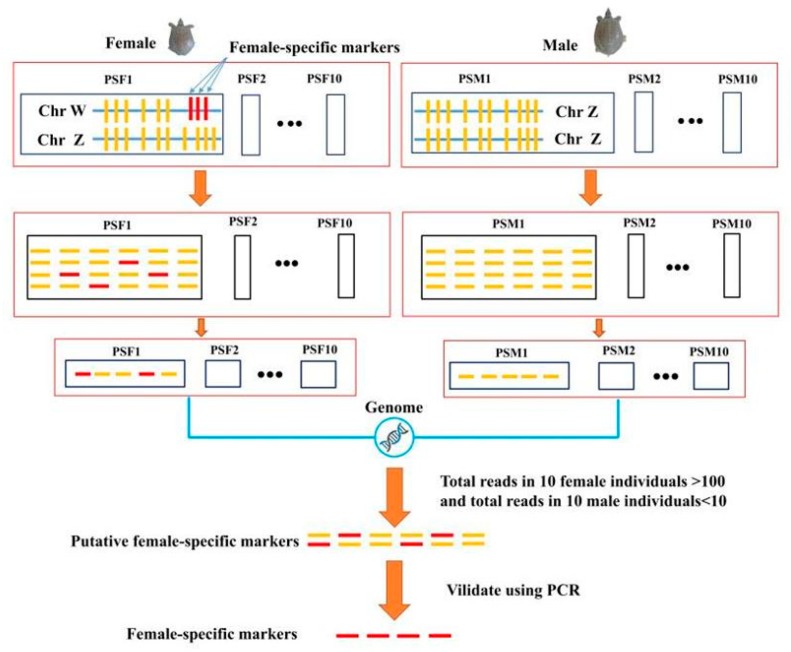
Workflow of sex-specific marker development in the *Pelodiscus sinensis* based on restriction site-associated DNA sequencing (RAD-seq).

The detailed workflow of the sex-specific marker development based on RAD-seq is shown in Figure 1. The RAD-seq generated 26.28 Gb of raw data from the 10 individual female libraries and 23.41 Gb of raw data from the 10 individual male libraries. The average raw data per turtle was 2.63 Gb for females and 2.34 Gb for males. The Q20 high-quality score for the data ranged from 90.72% to 91.20% and the Q30 score ranged from 90.71% to 92.68% in males, while Q20 ranged from 95.96% to 96.86% and the Q30 score ranged from 91.17% to 92.70% in females. The GC content ranged from 43.67% to 44.10% in male individuals and from 43.77% to 44.17% in female individuals. There were 200,108,964 raw reads for the male samples and 179,268,916 for the female samples. After filtering, 193,656,716 and 173,982,866 high-quality reads were obtained from male and female individuals, respectively. The details are shown in Table 2. The RAD-sequencing result is available via the NCBI database with the SRA accession number PRJNA530350. 

### 3.2. Identification of Sex Specific Regions and Molecular Marker Design 

Each read from the PSF and PSM individuals was compared with the reference genome sequence of the Chinese soft-shelled turtle (PelSin_1.0) at the NCBI. The alignment results are shown in Table 3. The alignment results identified 19,904 scaffold sequences that could be subjected to further analysis. Among them, 120 scaffolds (0.6%, 120/19,904) were extracted according to the criteria of total reads >100 in all ten female samples, but total reads <10 in all ten male samples. To validate the candidate female-specific markers, the top 10 scaffold sequences based on read number were identified as candidate sequences, in which the selectivity was as high as 0.05% to identify the sex-specific regions. 

### 3.3. Identification of Candidate Sex-Difference Regions 

**Figure 2:**
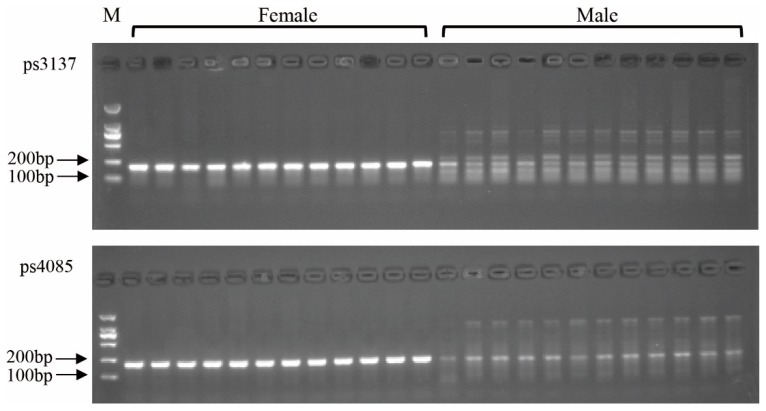
Detection marker ps4085 and ps3137 in male and female *Pelodiscus sinensis* populations.

Based on the above scaffolds alignment, the top 10 candidate sex-difference sequences were used to design primers (Table 1). After the PCR amplification and agarose gel detection, different amplification products were demonstrated to exist in male and female individuals for sequences 4085 and 3137 using primers ps4085 and ps3137 (Figure 2). However, these bands only displayed differences in band intensity between female and male individuals, which were not similar, as expected. 

### 3.4. Genome Walking

Although the agarose gel detection displayed different types of bands for the sequences of loci 4085 and 3137, their specific sequences were only about 100 bp, which were too short to design effective primers for PCR amplification. Thus, genome walking was performed to obtain longer sequences based on the known sequence information of 4085 and 3137. After eight rounds of genome walking amplification, four from walking to the right and four from walking to the left, longer sequences were amplified according to genomic fragments from the pool of female and male samples. Furthermore, each PCR-amplified product was sequenced 10 times to ensure their accuracy. Finally, a sequence of 5967 bp was obtained for locus 4085, while a sequence of 6532 bp was obtained for locus 3137, based on the PCR fragments from the female pools. Subsequently, the sex-specific markers ps4085s, ps3137s1, and ps3137s2 were designed (Table 1). 

### 3.5. Identification of Sex-Specific Markers

**Figure 3:**
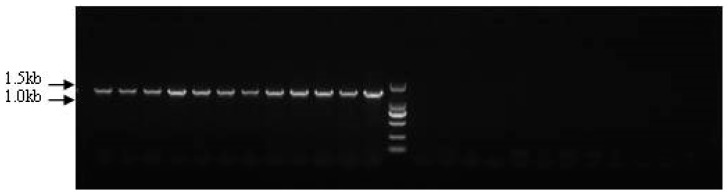
Detection of sex-specific marker ps4085s in male and female *Pelodiscus sinensis* populations.

Based on the genome walking results, we detected the sex-specific markers ps4085s, ps3137s1, and ps3137s2, which are located in loci 4085 and 3137. The three specific regions obtained by ps4085s, ps3137s1, and ps3137s2 were 1434 bp, 1313 bp, and 864 bp, and their GC contents were 56.45%, 51.33%, and 64.47%, respectively. The PCR amplification products were detected using 1.5% agarose gel electrophoresis. For marker ps4085s, the agarose gel only displayed application products (1434 bp) in the female individuals, and no bands were amplified from the male individuals (Figure 3). This indicated that ps4085s could be a sex-specific marker for sex identification in Chinese soft-shelled turtles. 

**Figure 4:**
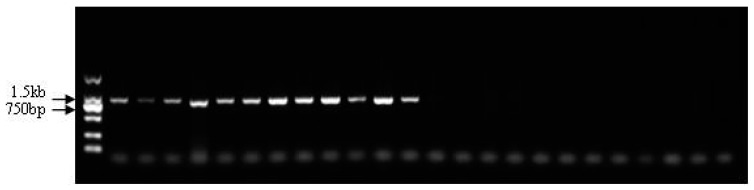
Detection of sex-specific marker ps3137s2 in male and female *Pelodiscus sinensis* populations.

**Figure 5:**
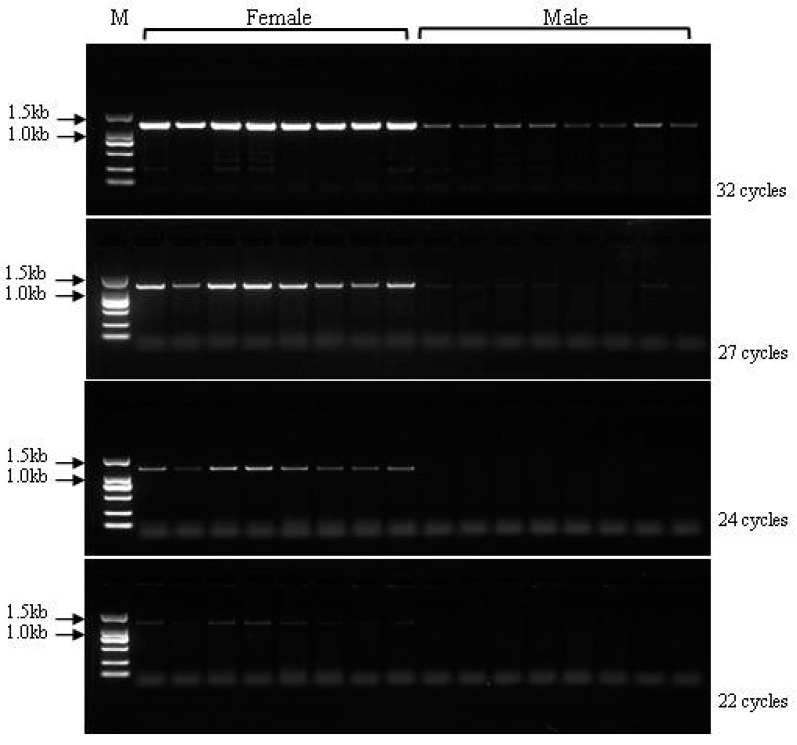
Detection of sex-specific marker ps3137s1 in male and female *Pelodiscus sinensis* populations.

For locus 3137, we observed deletion fragments in male individuals compared with female individuals after completing the genome walking sequencing. Based on the 6532 bp sequence of locus 3137, two pairs of primers (ps3137s1 and ps3137s2) located at non-overlapping regions were designed to detect the specific region. The application product (864 bp) using primer pair ps3137s2 identified only one clear band in the female individuals, but no bands were amplified from the male individuals (Figure 4). Interestingly, the amplification products (1313 bp) for marker ps3137s1 were observed in both female and male populations. However, the products exhibited a dosage effect between females and males, in which the amount of products in female Chinese soft-shelled turtles was higher than that in the males. To characterize and optimize the dosage difference between the female and male individuals, we used four PCR amplification reactions comprising 32, 27, 24, and 22 cycles. The results showed that the amount of PCR product decreased as the number of amplification cycles was reduced (Figure 5). The agarose gel showed that in the female individuals, the PCR product bands became very faint, whereas the bands in the male individuals disappeared when using less than 22 cycles. Thus, the optimal amplification conditions to distinguish females from males were set at 24 cycles. 

### 3.6. Validation of the Developed Markers

**Figure 6:**
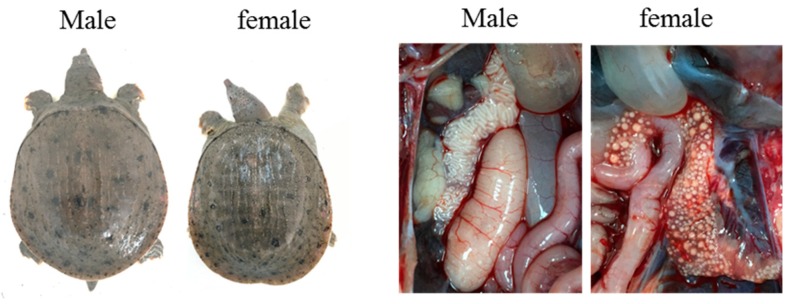
Morphology and gonad tissue observation in male and female *Pelodiscus sinensis*.

To further verify the universality of the developed sex-specific markers ps4085 and ps3137, two farmed populations of the Chinese soft-shelled turtles were collected from Xiantao (Hubei province) and Hanshou (Hunan province), respectively. Pictures of macroscopic morphology observation and gonad tissue are shown in Figure 6 and some of agarose gel results have been displayed as  supplementary material (Supplementary Figures). The detection sex identification comparison result using gonadal tissue observation and markers in two *Pelodiscus sinensis* populations is shown in Table 4. 

As expected, the fragment sizes in these individuals were identical to those in the farmed population of Anhui Province, in that the PCR amplification products were only observed in the female individuals, and no bands were found in the male individuals for ps4085 and ps3137. The identification results of gonadal tissue and sex-specific markers are presented in Table 4. The results indicated that these three sex-specific markers could identify the genetic sex of Chinese soft-shelled turtles.

## 4. Discussion 

To study sex differentiation and sex control, sex-specific DNA markers are required. Sex-specific marker development is also crucial for genetic breeding. Sexual dimorphism exists widely in animals, especially in the lower vertebrates, including fish, reptiles, and amphibians. Sex-specific genetic markers are often required to study sex-associated phenomena, such as significant sexual dimorphisms of growth rate and body size [19,20]. The development of next generation sequencing (NGS) technology has led to the emergence of restriction site-associated DNA sequencing (RAD-seq), in which sequences are obtained from the DNA flanking specific restriction sites throughout the whole genome, which can not only detect a huge number of genetic polymorphisms at a low cost, but also produces mass genetic markers, even if no genome information is available for a species [17,21]. Previously, most studies employed to identify sex-specific markers needed to construct linkage maps from test-crosses [22,23]. However, in many species, it is not feasible to generate test-crosses because they may have a long generation time, a small number of offspring, or are not easy to breed in captivity [24,25]. However, RAD-seq does not require test crossing, and thus is considered as a particularly powerful tool to explore genetic variation and identify sex-specific DNA-based (or molecular) genetic markers in ‘non-model’ species [12,14,21,26] 

In the present study, we isolated two female-specific DNA fragments using RAD-seq and subsequently identified a 5967 bp fragment from locus ps4085 and a 6532 bp fragment from locus ps3137 from the female-derived sequences using genomic walking. Finally, primer pairs were developed for three female-specific markers ps4085, ps3137s1, and ps3137s2, which were successfully validated as effective and stable sex-specific markers to identify the genetic sex of Chinese soft-shelled turtles. Among them, the female-specific markers ps4085 and ps3137s2 were only detected in female individuals. Male sex markers would be indicative of an XY system, whereas female sex markers would indicate a ZW system [26]. In addition, the identified female-related markers were heterozygous in female individuals and homozygous in male individuals, which suggested a ZW/ZZ female heterogametic sex determination system. Therefore, these female-specific markers confirmed the previous conclusion that the sex determination system of the Chinese soft-shelled turtle belongs to the ZZ/ZW heterogametic system [15,16]. 

Interestingly, the female-related marker ps3137s1 amplification products were detected in both male and female individuals; however, a dosage difference between females and males under different PCR amplification cycles was observed. Higher levels of the ps3137s1 amplification product were produced from female samples than from male samples. A female-related marker was discovered to produce higher dosage association in females than in males of *Pseudobagrus ussuriensis*, in which the sex determination was a male heterogametic XX/XY system. And the dosage effect was explained by the presence of two X chromosomes in females and one X chromosome in males [13]. In the present study, the female-specific marker primer pair ps3137s1F/ps3137s1R produced higher levels of amplification products in heterogametic ZW female individuals. Genomic variations, including deletions, insertions, inversions, and duplications, represent an important source of genetic diversity in organisms [27,28]. In addition, gene dosage effects are usually caused by sequence fragment deletions, duplications, or loss-of-function mutations [29]. In fact, when we sequenced the 6532 bp fragment of locus ps3137 in female and male individuals, we observed deletion fragments in the male individuals compared with those in the female individuals. Therefore, we speculated that marker ps3137s1 was located on a W sex chromosome, and not on an autosome. Thus, the dosage effect was probably caused by a difference in gene copy number caused by the identified deletions. 

In summary, we developed three female-specific DNA markers in the Chinese soft-shelled turtle using RAD-seq. These female-specific DNA markers could identify the genetic sex of Chinese soft-shelled turtle populations with a 100% accuracy. The developed markers will enable studies of sex determination and sex control breeding in Chinese soft-shelled turtles at all life stages.

## Figures and Tables

**Table 1:** Candidate sex-specific markers used in female Chinese soft-shelled turtles.

Locus	Markers	Sequence (5′−3′)	Purpose
ps4085	ps4085F	GTTTGAAGTGCTGCTGGGAAG	Identification of the candidate female-specific marker region
	ps4085R	TTCCCCGTATAAAGCCAGGG	
ps3137	ps3137F	CACGGGGTGAAGCACTCATT	
	ps3137R	TGTCTCACCTCCTTTGCCTC	
ps2962	ps2926F	AGAATTCATGCGGTTACTCAACT	
	ps2926R	GAGGCAGCATTTGACGGGTA	
ps5510	ps5510F	GGGAAATAACGGGACCCCAG	
	ps5510R	CCACTGGTATCTGGCCACTC	
ps5388	ps5388F	GGAATTCTGCGGGTAGGAGG	
	ps5388R	CCTAACTCCTGGGGGAACCT	
ps7307	ps7307F	GCCAGGACACTGGCATTAAC	
	ps7307R	GAGGTCCTGGACACCTCTGA	
ps7885	ps7885F	CTCCATAGGGGAGCACAAGC	
	ps7885R	TGCAGGTGCAGAACCACTTA	
ps7837	ps7837F	GGTCTTAATGCACCCCGTTG	
	ps7837R	ACATTTCCCACCCCTTGGTC	
ps5946	ps5946F	TTAGGTGCCTAGTGCCAAGC	
	ps5946R	CCCGTCCCGAAGACTGTATG	
ps6704	ps6704F	CCCAACTGCATTCCCGTTCT	
	ps6704R	CCCTGGACGTGGAAAACTCA	
ps4085s	ps4085sF	GCTGCTGGGAAGACTTAGAGTTG	Identification of sex-specific marker
	ps4085sR	CTTGAATGTGACTAGGAAGGCTTC	
ps3137s1	ps3137s1F	TGACTGGCACTGGAGAAGGA	
	ps3137s1R	GCATCAACCACAGCCCTACA	
ps3137s2	ps3137s2F	TCCAGGGCAGAAAGACACTC	
	ps3137s2R	ATGTCCCGGTGGATGGC	

**Table 2:** Raw data information from RAD-Seq.

Sample	Raw Data bp	Raw Reads Num.	Q20%	Q30%	GC%	HQ Reads Num (%)
PSM1	2,957,269,944	21,351,164	96.46	92.10	43.67	20,687,143 (96.89%)
PSM2	2,411,667,340	16,504,674	96.89	92.68	43.32	16,120,788 (97.67%)
PSM3	2,595,681,148	17,558,578	96.07	91.17	43.98	16,952,904 (96.55%)
PSM4	2,226,436,872	15,037,496	95.88	90.87	44.43	14,450,386 (96.10%)
PSM5	2,387,165,348	16,221,390	96.14	91.32	44.47	15,691,924 (96.74%)
PSM6	2,054,437,868	13,875,546	95.94	90.87	43.82	13,385,482 (96.47%)
PSM7	2,481,263,740	16,868,546	96.22	91.44	44.07	16,346,936 (96.91%)
PSM8	2,001,024,316	13,705,646	96.55	92.02	44.33	13,397,652 (97.75%)
PSM9	1,987,020,528	13,411,628	95.86	90.84	44.43	12,877,632 (96.02%)
PSM10	2,307,425,680	15,574,296	95.85	90.72	44.10	14,991,562 (96.26%)
PSF1	2,100,080,936	14,384,116	96.62	92.28	44.17	14,034,716 (97.57%)
PSF2	2,467,693,044	16,759,374	96.44	91.92	43.89	16,275,792 (97.11%)
PSF3	2,213,291,672	15,159,532	96.80	92.60	43.95	14,830,036 (97.83%)
PSF4	2,201,335,428	14,891,202	96.28	91.59	43.88	14,393,778 (96.66%)
PSF5	2,714,080,420	18,338,706	95.96	91.17	44.06	17,563,492 (95.77%)
PSF6	3,250,793,660	22,265,710	96.77	92.52	43.89	21,795,770 (97.89%)
PSF7	2,429,666,592	16,641,552	96.72	92.46	44.02	16,261,182 (97.71%)
PSF8	3,582,429,740	24,537,190	96.13	91.90	43.89	23,398,702 (95.36%)
PSF9	2,650,642,556	18,155,086	96.86	92.70	43.83	17,784,484 (97.96%)
PSF10	2,673,816,280	18,136,448	96.45	91.86	43.77	17,644,914 (97.29%)

Note: RAD-seq, restriction site-associated DNA sequencing (RAD-seq); Q20, quality scores of ≥20; Q30, quality scores of ≥30.

**Table 3:** RAD-seq reads mapped to the Chinese soft-shelled turtle genome (assembly PelSin_1.0).

Sample	All Reads	Single Mapped Reads	Paired Mapped Reads	Unmapped Reads	Alignment Ratio (%)
PSM1	20,687,143	154,583	12,937,651	422,017	97.96
PSM2	16,120,788	52,145	7,866,467	335,709	97.92
PSM3	16,952,904	124,324	8,213,599	401,382	97.63
PSM4	14,450,386	108,720	7,007,918	325,830	97.75
PSM5	15,691,924	121,822	7,592,756	384,590	97.55
PSM6	13,385,482	98,822	6,511,777	263,106	98.03
PSM7	16,346,936	116,368	7,961,448	307,672	98.12
PSM8	13,397,652	97,082	6,530,704	239,162	98.21
PSM9	12,877,632	99,272	6,224,305	329,750	97.44
PSM10	14,991,562	113,334	7,283,306	311,616	97.92
PSF1	14,034,716	910,48	6,857,225	229,218	98.37
PSF2	16,275,792	106,920	7,934,999	298,874	98.16
PSF3	14,830,036	92,616	7,229,123	279,174	98.12
PSF4	14,393,778	92,498	7,026,635	248,010	98.28
PSF5	17,563,492	121,625	8,549,820	342,227	98.05
PSF6	21,795,770	142,215	10,640,276	373,003	98.29
PSF7	16,261,182	107,657	7,921,154	311,217	98.09
PSF8	23,398,702	138,420	11,436,319	387,644	98.34
PSF9	17,784,484	109,638	8,697,230	280,386	98.42
PSF10	17,644,914	112,739	8,617,538	297,099	98.32

**Table 4:** Comparison result of sex identification using gonadal tissue observation and markers in two *Pelodiscus sinensis* populations.

Name	Size	Frequency (%)			
		Hanshou Population		Xiantao Population	
		Female	Male	Female	Male
ps4085	1434 bp	48/48 (100)	48/48 (100)	100/100 (100)	100/100 (100)
ps3137s1	1313 bp	48/48 (100)	48/48 (100)	100/100 (100)	100/100 (100)
ps3137s2	864 bp	48/48 (100)	48/48 (100)	100/100 (100)	100/100 (100)

## Supplementary Materials

The following are available online at https://www.mdpi.com/2073-4425/10/4/302/s1, Figure S1: Detection sex-specific marker ps4085s in Hanshou *Pelodiscus sinensis* population. Figure S2A: Detection sex-specific marker ps4085s in Xiantao *Pelodiscus sinensis* population. Figure S2B: Detection sex-specific marker ps4085s in Xiantao *Pelodiscus sinensis* population. Figure S3: Detection sex-specific marker ps3137s2 in Hanshou Pelodiscus sinensis population. Figure S4A: Detection sex-specific marker ps3137s2 in Xiantao *Pelodiscus sinensis* population. Figure S4B: Detection sex-specific marker ps3137s2 in Xiantao *Pelodiscus sinensis* population. Figure S5:Detection sex-specific marker ps3137s1 in Hanshou *Pelodiscus sinensis* population. Figure S6A: Detection sex-specific marker ps3137s1 in Xiantao *Pelodiscus sinensis* population. Figure S6B: Detection sex-specific marker ps3137s1 in Xiantao *Pelodiscus sinensis* population.
