# Supplementary figures and images for: Development and Validation of Sex-Specific Markers in Pelodiscus Sinensis Using Restriction Site-Associated DNA Sequencing
Source: Genes (Basel). 2019 Apr 15;10(4):302. doi: 10.3390/genes10040302 (PMC6523797; doi:10.3390/genes10040302)

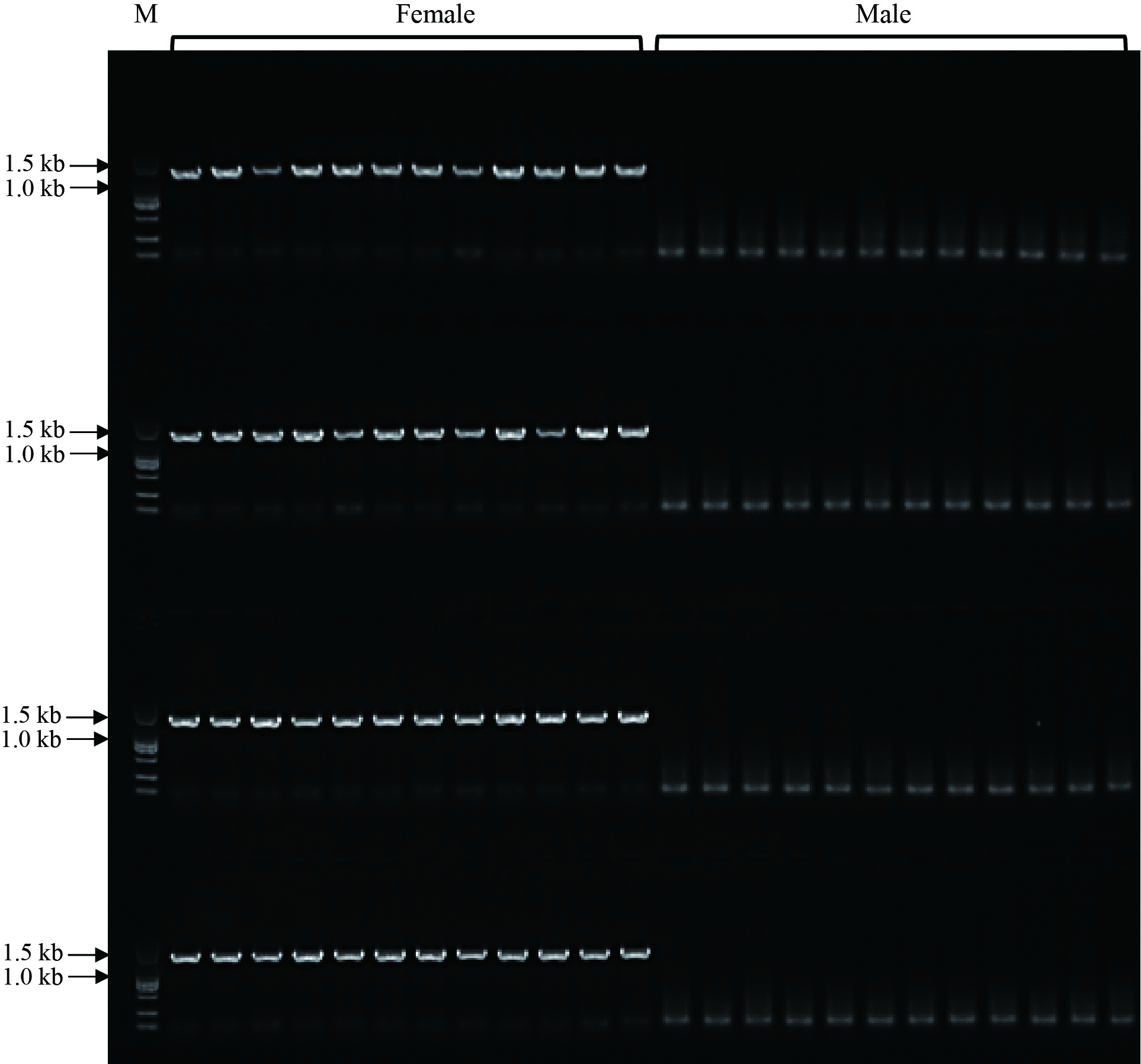

Supplement: Supplementary file 1 [file genes-10-00302-s001.zip › Supplemental figures/Supplement Figure 1.jpg]

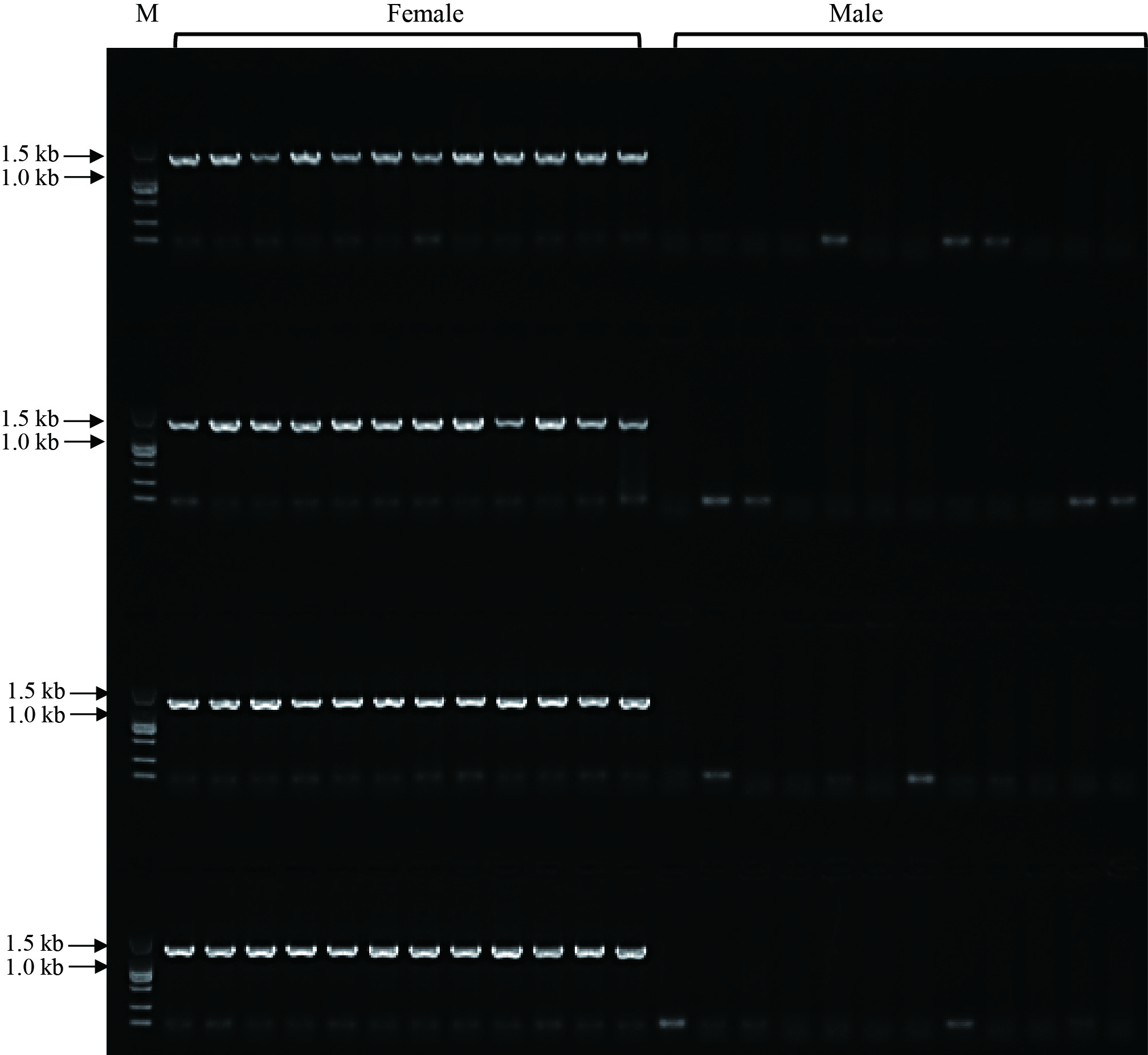

Supplement: Supplementary file 1 [file genes-10-00302-s001.zip › Supplemental figures/Supplement Figure 2A.jpg]

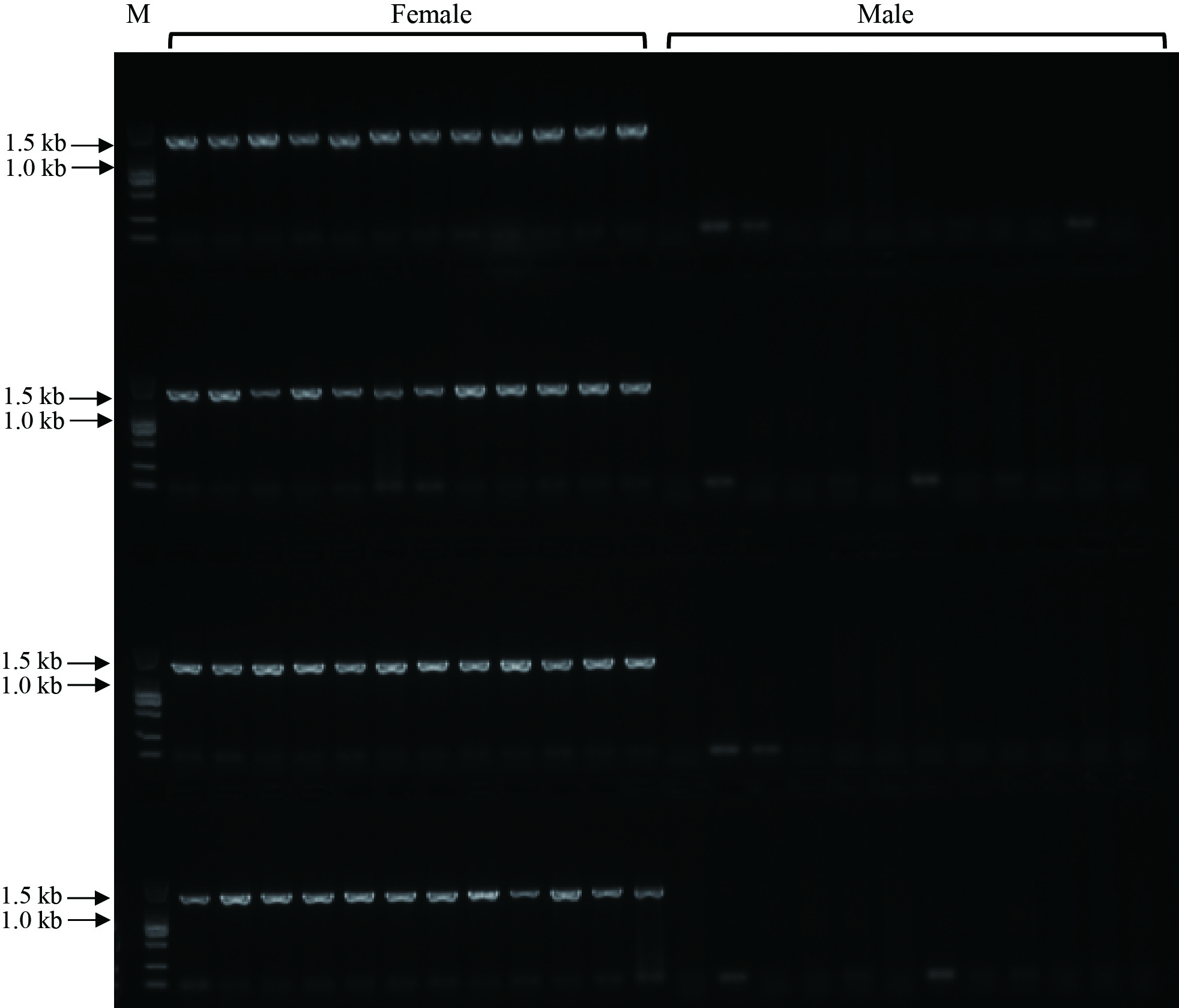

Supplement: Supplementary file 1 [file genes-10-00302-s001.zip › Supplemental figures/Supplement Figure 2B.jpg]

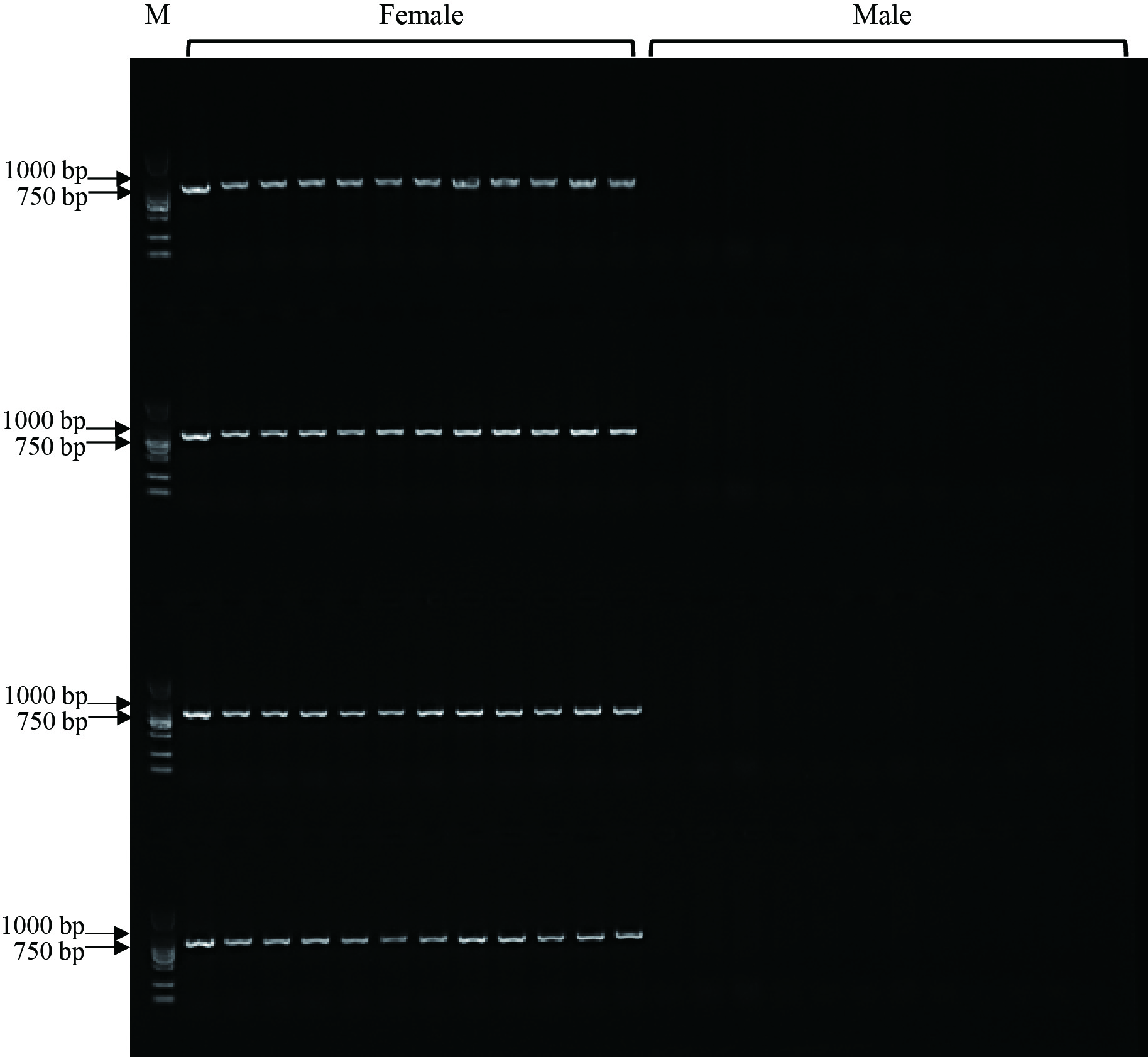

Supplement: Supplementary file 1 [file genes-10-00302-s001.zip › Supplemental figures/Supplement Figure 3.jpg]

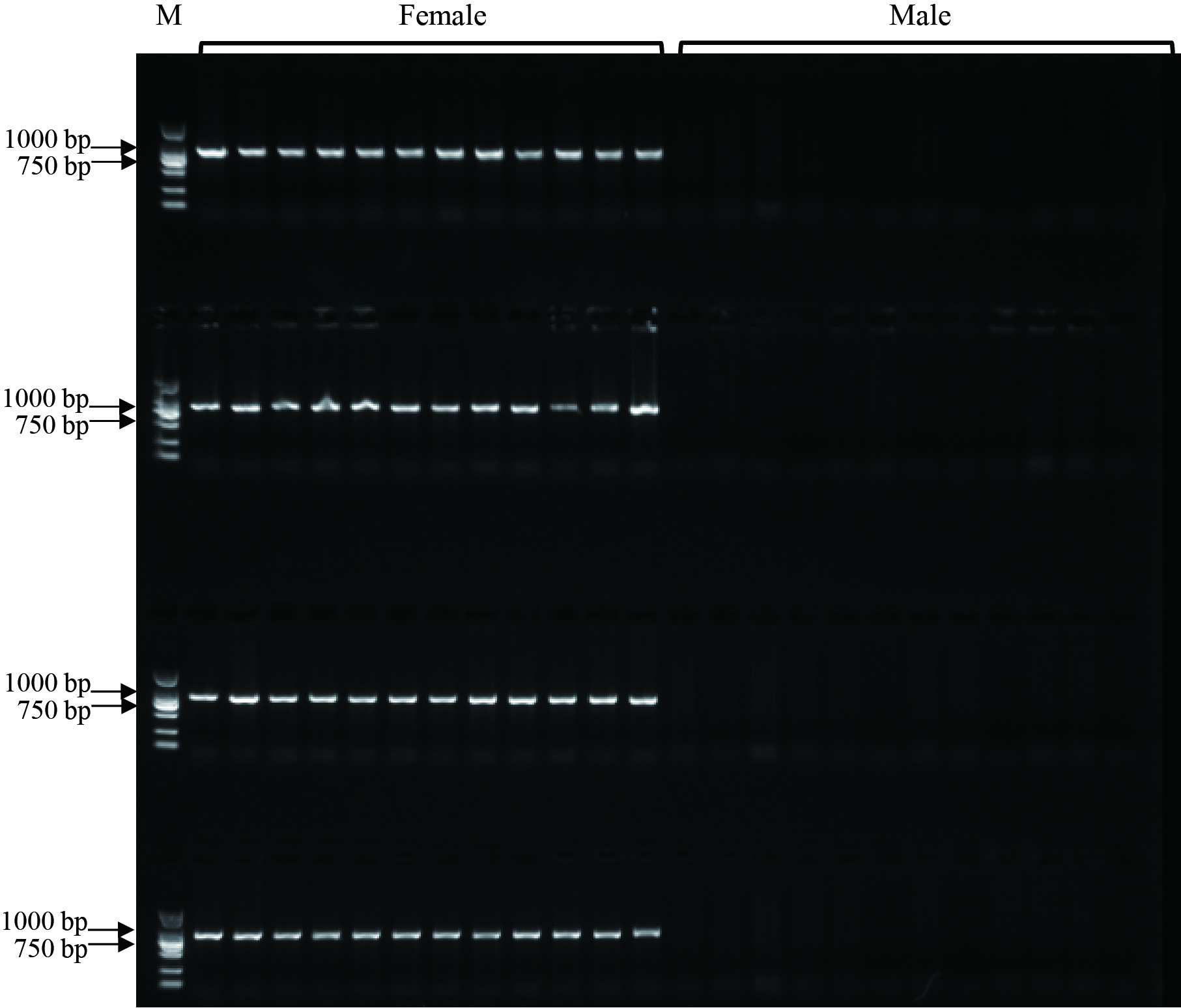

Supplement: Supplementary file 1 [file genes-10-00302-s001.zip › Supplemental figures/Supplement Figure 4A.jpg]

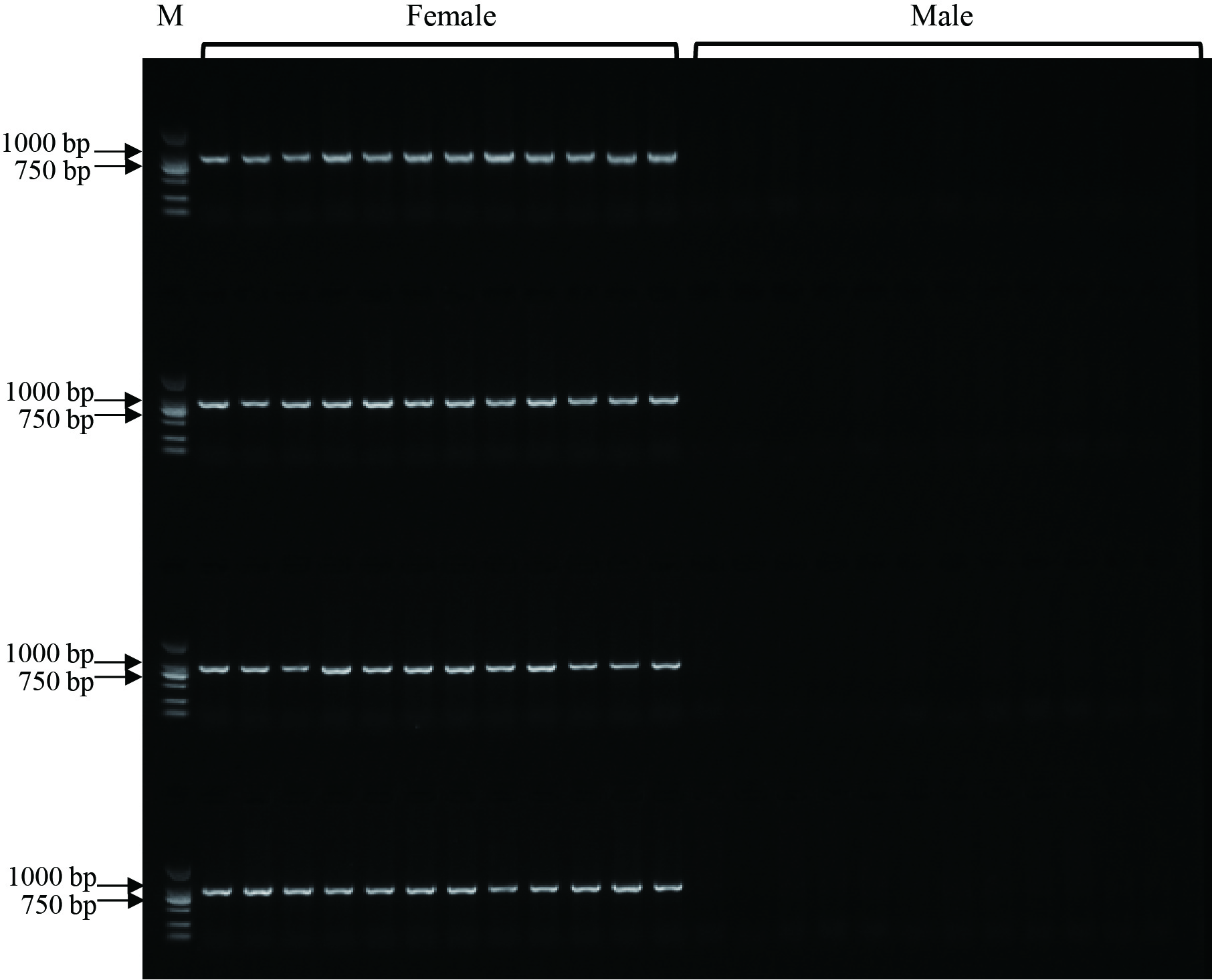

Supplement: Supplementary file 1 [file genes-10-00302-s001.zip › Supplemental figures/Supplement Figure 4B.jpg]

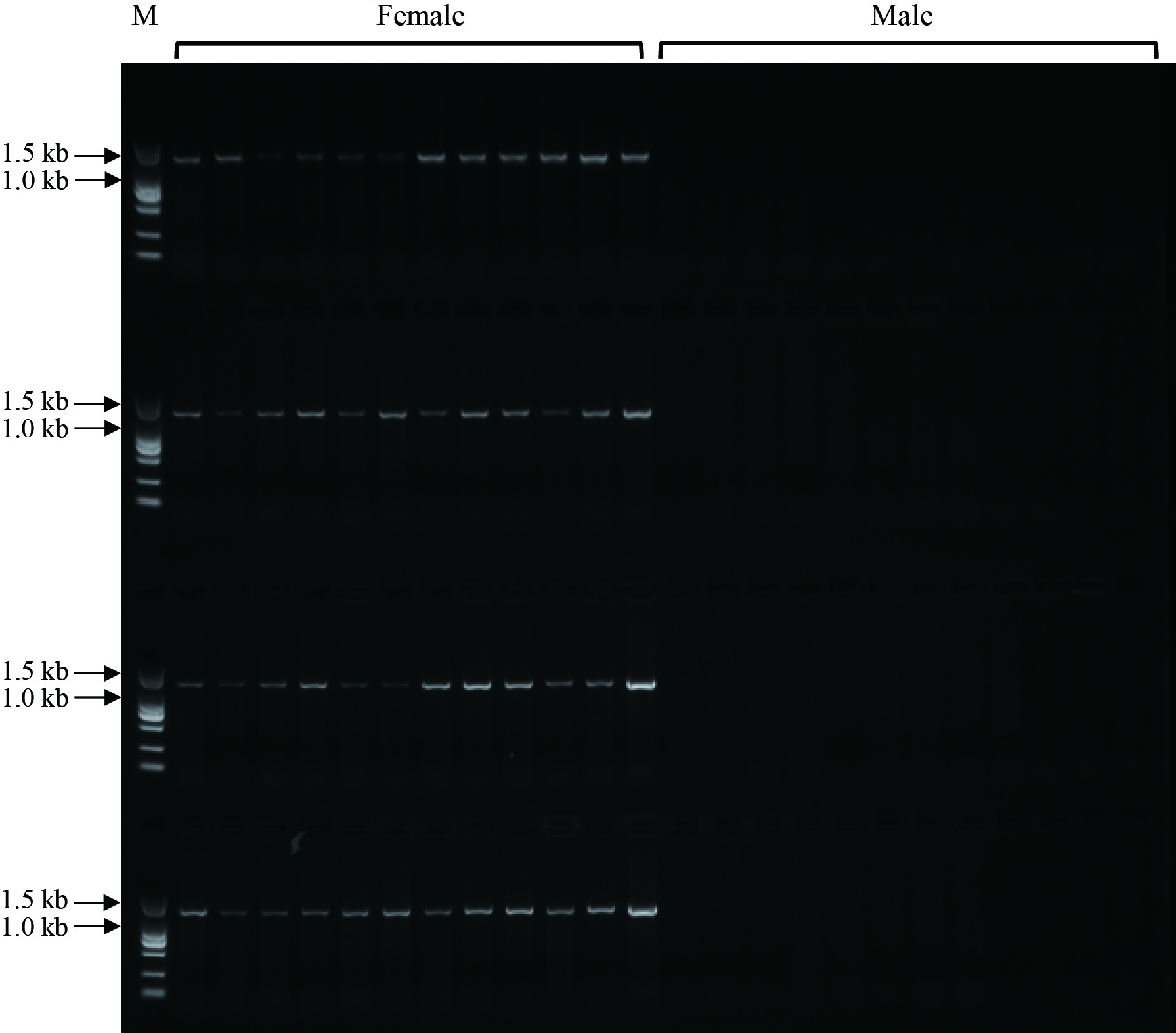

Supplement: Supplementary file 1 [file genes-10-00302-s001.zip › Supplemental figures/Supplement Figure 5.jpg]

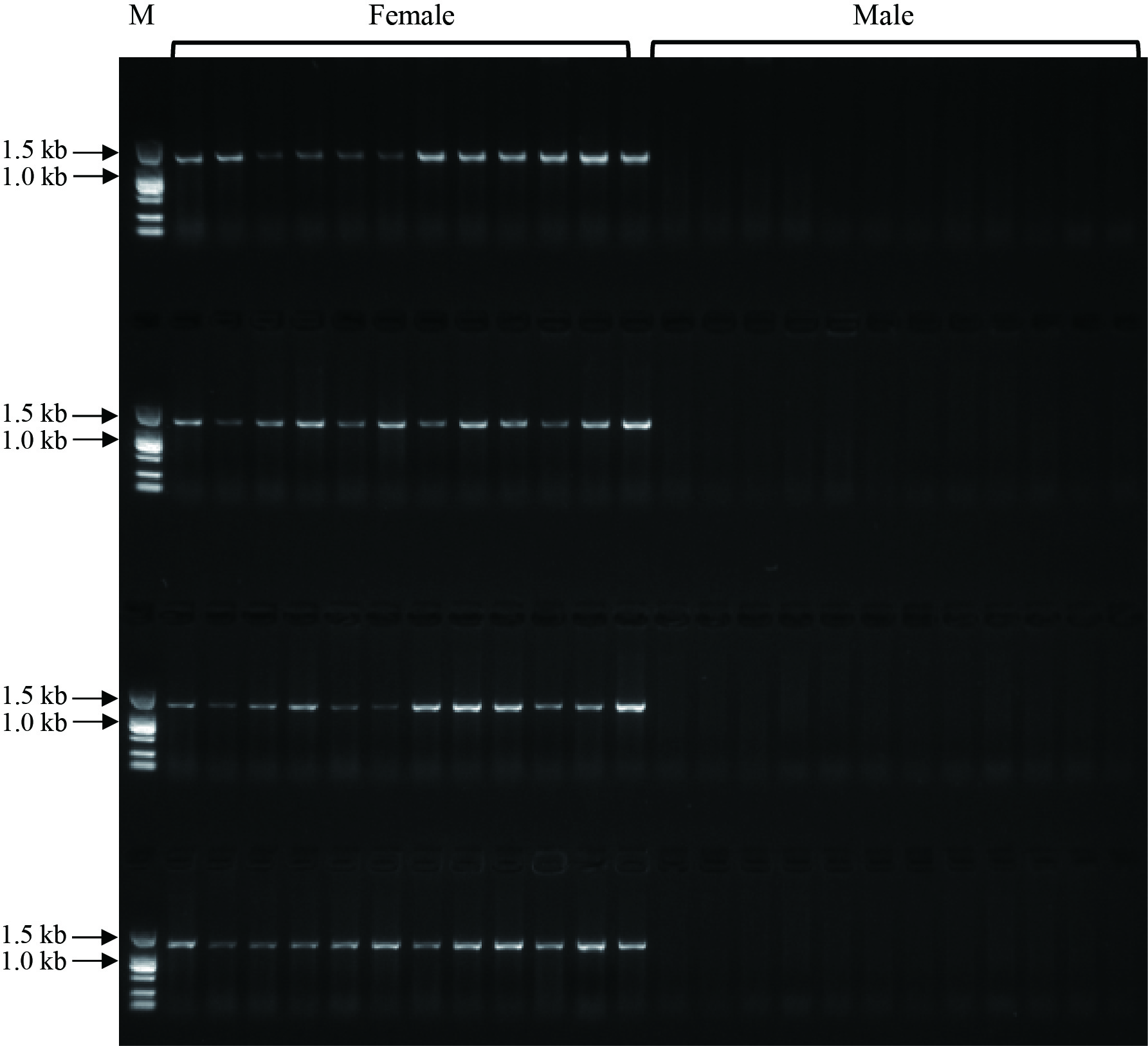

Supplement: Supplementary file 1 [file genes-10-00302-s001.zip › Supplemental figures/Supplement Figure 6A.jpg]

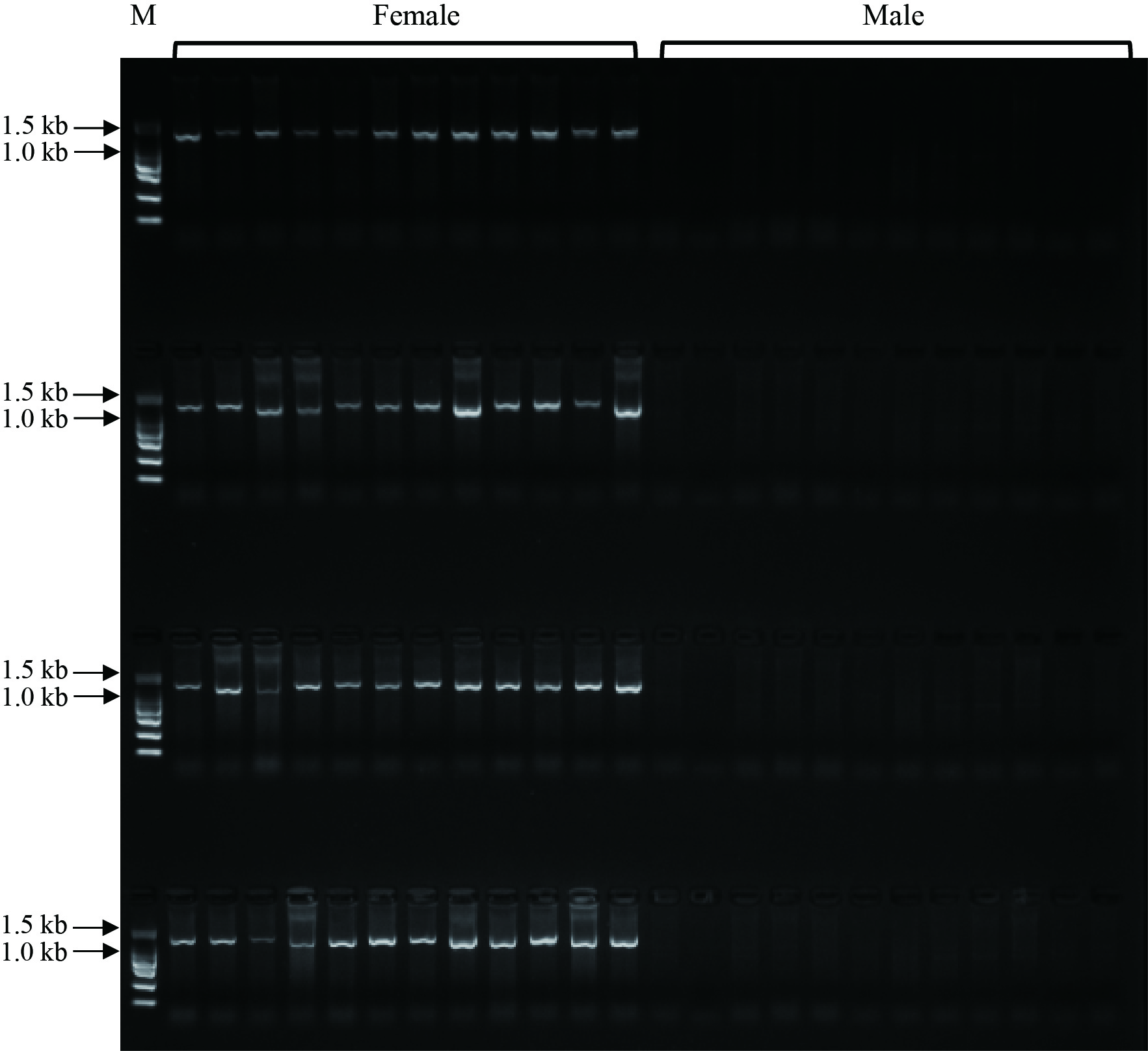

Supplement: Supplementary file 1 [file genes-10-00302-s001.zip › Supplemental figures/Supplement Figure 6B.jpg]
